# Fluctuation between Fasting and 2-H Postload Glucose State Is Associated with Glomerular Hyperfiltration in Newly Diagnosed Diabetes Patients with HbA1c < 7%

**DOI:** 10.1371/journal.pone.0111173

**Published:** 2014-10-31

**Authors:** Xinguo Hou, Chuan Wang, Shaoyuan Wang, Weifang Yang, Zeqiang Ma, Yulian Wang, Chengqiao Li, Mei Li, Xiuping Zhang, Xiangmin Zhao, Yu Sun, Jun Song, Peng Lin, Kai Liang, Lei Gong, Meijian Wang, Fuqiang Liu, Wenjuan Li, Fei Yan, Junpeng Yang, Lingshu Wang, Meng Tian, Jidong Liu, Ruxing Zhao, Shihong Chen, Li Chen

**Affiliations:** 1 Department of Endocrinology of Qilu Hospital, Shandong University, Jinan, Shandong, China; 2 Lukang Hospital of Jining, Jining, Shandong, China; 3 China National Heavy Duty Truck Group Corporation Hospital, Jinan, Shandong, China; 4 Department of Endocrinology, Second People's Hospital of Jining, Jining, Shandong, China; 5 Shantui Community Health Center, Jining, Shandong, China; 6 Department of Endocrinology, the Second Hospital of Shandong University, Jinan, Shandong, China; Institute of Endocrinology and Metabolism, Islamic Republic of Iran

## Abstract

**Objective:**

To investigate whether fluctuations between the fasting and 2-h postload glucose ([2-hPBG]-fasting blood glucose [FBG]) states are associated with glomerular hyperfiltration (GHF) in middle-aged and elderly Chinese patients with newly diagnosed diabetes.

**Design and Methods:**

In this study, we included 679 newly diagnosed diabetes patients who were ≥40 years old. All the subjects were divided into two groups; those with HbA1c<7% and ≥7%. The Chronic Kidney Disease Epidemiology Collaboration (CKD-EPI) equation was used to estimate the glomerular filtration rate (GFR). GHF was defined as an eGFR ≥ the 90th percentile. First, a multiple linear regression analysis was used to estimate the association of 2-hPBG-FBG with eGFR. Then, a generalized additive model was used to explore the possible nonlinear relationship between 2-hPBG-FBG and eGFR. Next, the 2-hPBG-FBG values were divided into four groups as follows: 0–36, 36–72, 72–108 and ≥108 mg/dl. Finally, a multiple logistic regression analysis was used to investigate the association of 2-hPBG-FBG with the risk of GHF.

**Results:**

For the group with HbA1c<7%, the eGFR and the percentage of GHF were significantly higher compared with the group with HbA1c≥7%. After adjusting for age, gender, body mass index (BMI), systolic blood pressure (BP), diastolic BP, fasting insulin, cholesterol, triglycerides, smoking, drinking and glycated hemoglobin (HbA1c), 2-hPBG-FBG was significantly associated with increased eGFR and an increased risk of GHF (the GHF risk increased by 64.9% for every 36.0 mg/dl [2.0 mmol/L] 2-hPBG-FBG increase) only in those patients with HbA1c<7%. Additionally, 2-hPBG-FBG and eGFR showed a nonlinear association (*P*<0.001).

**Conclusions:**

Increased fluctuations between the fasting and 2-h postload glucose states are closely associated with increased eGFR and an increased risk of GHF in newly diagnosed diabetes patients with HbA1c<7%.

## Introduction

Diabetic nephropathy (DN), which is characterized by an initial period of glomerular hyperfiltration (GHF) followed by progressively increasing proteinuria and a gradual decline in the glomerular filtration rate (GFR), is the leading cause of chronic kidney disease (CKD) and is associated with increased cardiovascular mortality in diabetic patients worldwide [1], [2]. As a functional change occurring during the early stage of diabetes, GHF is likely to correlate with the progression of DN and may even contribute to the initiation of this disease [3], [4]. The prevalence of GHF reported in type 2 diabetic patients has varied widely from 7 to 73% [5], [6], [7], [8]. Therefore, the screening for risk factors of GHF during the early stage of type 2 diabetes is critical for DN prevention.

Hyperglycemia is closely related to the development of the vascular complications of diabetes and can be prevented by the strict control of blood glucose [9], [10]. As a relatively objective indicator of average blood glucose within the past two or three months, glycated hemoglobin (HbA1c) has been used as the standard for glucose control. However, the incidence of diabetic complications increases more rapidly in proportion to postprandial glucose or to peak glucose levels compared with average blood glucose levels, indicating the important role of blood glucose fluctuations in the development of diabetic complications [11], [12], [13], [14]. A growing body of research has suggested that glucose fluctuations may accelerate the renal complications associated with diabetes independent of the presence of hyperglycemia; however, the results are inconsistent. One study of type 1 diabetes patients has revealed that glucose fluctuations do not predict the development of nephropathy [15]. In contrast, two other studies have indicated that glucose fluctuations may affect the severity of CKD in patients with type 2 diabetes [16], [17]. Therefore, it is necessary to further clarify the influence of glucose fluctuations on the changes in renal function that occur in association with diabetes, particularly during the early stage of the disease (as indicated by GHF).

However, the definition of glucose fluctuation is currently uncertain, and the monitoring of glucose fluctuation is complicated in clinical practice and large epidemiological studies. Because the most common glucose fluctuations occur following meals, differences between the fasting and postprandial glucose levels may reflect the state of glucose fluctuation (to some extent). Therefore, it should be determined whether these differences are related to GHF. However, the total calorie intake often differs between subjects because of dietary variations. To standardize calorie intake to evaluate glycemic control, we performed the 75-g oral glucose tolerance test (OGTT) in a representative sample of the Chinese population and used 2-h postload blood glucose (2-hPBG) to represent the postprandial glucose levels. We investigated whether the fluctuations between fasting blood glucose (FBG) and 2-hPBG-FBG were associated with GHF in middle-aged and elderly Chinese patients newly diagnosed with diabetes.

## Materials and Methods

### Ethics statement

This work was performed as part of the baseline survey of the REACTION study assessing the association of diabetes and cancer, which included 259,657 adults (40 years of age and older) from 25 communities across mainland China from 2011 to 2012 [18]. This study was approved by the Ruijin Hospital Ethics Committee of the Shanghai Jiao Tong University School of Medicine. Written informed consent was obtained from all participants.

### Study population

For this screening study, 10,028 subjects were randomly recruited who were ≥40 years old and resided in the Shandong province from January 2012 to April 2012. A 75-g OGTT was performed for all participants. We first selected the 2044 subjects whose 75-g OGTT results were indicative of diabetes (see below). Then, 1181 previously diagnosed diabetic patients were excluded based on their medical histories. Further exclusion criteria were as follows: (1) lower 2-hPBG than FBG levels; (2) missing data for the calculation of the eGFR; (3) previously diagnosed kidney disease, including autoimmune or drug-induced kidney disease, nephritis, renal fibrosis or renal failure, or prior kidney transplant and current dialysis treatment; (4) previously diagnosed hepatic disease, including fatty liver, liver cirrhosis and autoimmune hepatitis; and (5) any malignant disease. A total of 679 subjects (423 women) were eligible for the analysis.

### Data collection

Demographic characteristics, lifestyle information and previous medical histories were obtained by trained investigators through a standard questionnaire. BMI was calculated as weight (kg) divided by height squared (m^2^). Blood pressure (BP) was measured 3 times consecutively (OMRON Model HEM-752 FUZZY, Omron Company, Dalian, China), and the average reading was used for the analysis. After an overnight fast, venous blood samples were collected for measurements of FBG, fasting insulin, cholesterol, triglycerides and creatinine. 2-hPBG was measured after subjects had completed the 75-g OGTT. HbA1c was measured by high-performance liquid chromatography (VARIANT II and D-10 Systems, BIO-RAD, USA). The homeostasis model assessment of insulin resistance (HOMA-IR) index was calculated as follows: fasting insulin concentration (mIU/L)×FPG concentration (mmol/L)/22.5 [19]. The eGFR was calculated from the creatinine level using the following formulas according to the Chronic Kidney Disease Epidemiology Collaboration (CKD-EPI) [20].

### Definition

There is no standard definition of hyperfiltration. Previous studies have arbitrarily used eGFR thresholds of 125 to 140 mL/min/1.73 m^2^ as the defining criteria for hyperfiltration. However, GFRs decrease with age [21]. The present study contained only middle-aged and elderly subjects, who were expected to present with relatively low eGFRs. Therefore, GHF was defined as an eGFR ≥ the 90th percentile (101.81 mL/min/1.73 m^2^) in this study.

Newly diagnosed diabetes has been defined by the World Health Organization (WHO) in 1999 [22] as FBG≥126 mg/dl (7.0 mmol/L) and/or 2-hPBG≥200 mg/dl (11.1 mmol/L) without a history of diabetes. For newly diagnosed diabetes patients who are not receiving antidiabetic drug therapy, HbA1c levels may be reflective of the clinical course of diabetes. Therefore, we divided all subjects into two groups according to the target value of HbA1c [23] as follows: patients with HbA1c<7% were considered to be at the very early stage, and those with HbA1c ≥7% were considered to be at a comparably later stage.

### Statistical analysis

Normally distributed continuous variables are expressed as the mean±SD, and variables with non-normal distributions are presented as the median (interquartile range). Categorical variables are presented as numbers (%). The differences between the HbA1c groups were detected by Student’s *t* test (normally distributed continuous variables), Mann-Whitney U test (skewed continuous variables), or chi-square test (categorical variables). After verifying the assumption of a linear relationship between the dependent and independent variables that were introduced into the linear regression model (assessed using a histogram of the residuals, together with a scatterplot of the standardized residuals versus the standardized predicted values in the different models), a multiple linear regression analysis was used to estimate the association of 2-hPBG-FBG with eGFR. Three models were constructed as follows: the first was not adjusted; the second was adjusted for age, gender, BMI, systolic BP and diastolic BP; and the third was adjusted for age, gender, BMI, systolic BP, diastolic BP, Log (fasting insulin), cholesterol, Log (triglyceride), smoking, drinking and HbA1c. We used a generalized additive model to explore the possible nonlinear association of 2-hPBG-FBG with eGFR after adjusting for the aforementioned factors. The 25th, 50th, and 75th percentiles of 2-hPBG-FBG were 37.8 mg/dl (2.1 mmol/L), 75.6 mg/dl (4.2 mmol/L) and 108 mg/dl (6.0 mmol/L), respectively. To facilitate the use of the 2-hPBG-FBG values in clinical practice, they were divided into four groups according to 36 mg/dl (2.0 mmol/L) intervals as follows: 0–36, 36–72, 72–108 and ≥108 mg/dl. Then, the association of 2-hPBG-FBG (the four groups of 2-hPBG-FBG were introduced as ordinal dummy variables) with the risk of GHF was estimated using a multiple logistic regression analysis for the same three models. *P*<0.05 was considered statistically significant. The analysis of the generalized additive models was performed using Empower Stats 2.13.0. The data were analyzed using SPSS 16.0 (SPSS Inc., Chicago. IL).

## Results

### Characteristics of study participants

We included 679 subjects (423 women) with newly diagnosed diabetes who were divided into two groups based on HbA1c, using 7% as the cut-off value. As shown in Table 1, BMI, FBG, 2-hPBG, 2-hPBG-FBG, HbA1c, HOMA-IR, triglycerides, creatinine and the percentages of smoking and drinking subjects were significantly lower in the group with HbA1c<7%. In contrast, the eGFR and the percentage of GHF were higher compared with the subjects with HbA1c≥7%.

**Table 1:** Characteristics of study participants grouped by HbA1c category.

Characteristics	Total n = 679	HbA1c (%) <7 n = 364	HbA1c (%) ≥7 n = 315	*P*-value
Female (%)	423 (62.3%)	236 (64.8%)	187 (59.4%)	0.142
Age (years)	60.58±9.38	60.75±9.72	60.39±8.99	0.611
BMI (kg/m^2^)	27.09±3.35	26.80±3.40	27.44±3.27	**0.014**
Systolic BP (mmHg)	146.61±20.27	146.41±20.33	146.85±20.22	0.778
Diastolic BP (mmHg)	82.52±12.38	82.83±12.44	82.16±12.32	0.480
FBG (mg/dl)	150.87±45.21	132.56±25.23	172.03±53.30	**<0.001**
2-hPBG (mg/dl)	231.54±73.19	203.17±51.87	264.33±80.29	**<0.001**
2-hPBG-FBG (mg/dl)	80.67±48.73	70.60±47.91	92.30±47.13	**<0.001**
HbA_1c_ (%)	7.32±1.69	6.23±0.47	8.59±1.71	**<0.001**
Fasting insulin (mIU/L)	9.60 (6.60–13.60)	10.00 (6.83–13.70)	9.20 (6.30–13.50)	0.324
HOMA-IR index	3.43 (2.34–4.91)	3.20 (2.16–4.55)	3.81 (2.62–5.73)	**<0.001**
Cholesterol (mg/dl)	218.91±40.29	217.29±39.68	220.78±40.97	0.260
Triglycerides (mg/dl)	140.87 (99.68–198.46)	132.01 (95.68–179.86)	155.05 (111.64–217.07)	**<0.001**
Smoking (%)	97 (14.3%)	41 (11.3%)	56 (17.8%)	**0.016**
Drinking (%)	124 (18.3%)	54 (14.8%)	70 (22.2%)	**0.013**
Creatinine (mg/dl)	0.77±0.14	0.76±0.13	0.78±0.14	**0.012**
eGFR (mL/min/1.73 m^2^)	85.10±14.68	86.17±14.51	83.87±14.81	**0.042**
GHF (%)	68 (10.0%)	46 (12.6%)	22 (7%)	**0.014**

Data are presented as the means ± SD or as numbers (%). BMI, body mass index; BP, blood pressure; FBG, fasting blood glucose; 2-hPBG, 2-h postload blood glucose; HOMA-IR, homeostasis model assessment of insulin resistance; eGFR, estimated glomerular filtration rate; GHF, glomerular hyperfiltration.

### 2-hPBG-FBG is closely associated with eGFR in newly diagnosed diabetes patients with HbA1c<7%

**Figure 1:**
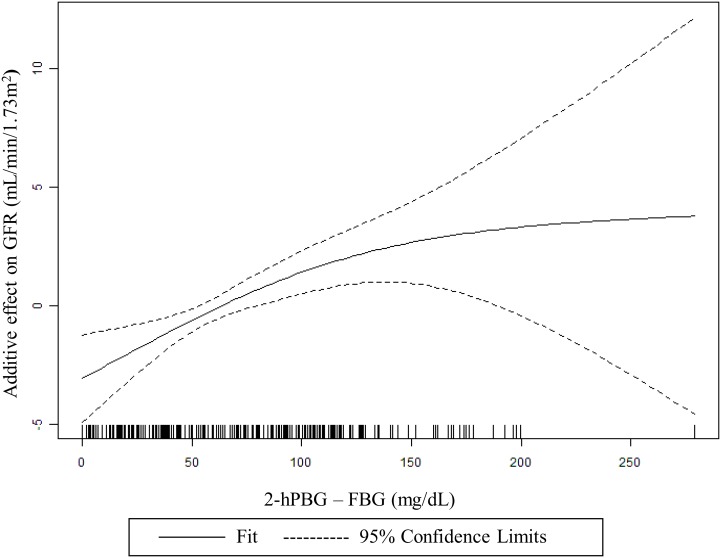
Nonlinear association of the difference between 2-h postload blood glucose and fasting blood glucose (2-hPBG-FBG) in association with estimated glomerular filtration rate (eGFR). The association was analyzed in a generalized additive model (df = 3, *P*<0.001) adjusted for age, gender, BMI, systolic BP, diastolic BP, fasting insulin, cholesterol, triglycerides, smoking, drinking and HbA1c.

As shown in Table 2, we constructed three models to analyze the association of 2-hPBG-FBG with eGFR (the calculation of HOMA-IR and 2-hPBG-FBG both involved FBG, so we adjusted for fasting insulin rather than for HOMA-IR in model 3). The linearity of the relationship was assessed using a histogram of the residuals for each model together with a scatterplot of the standardized residuals versus the standardized predicted values, which indicated an approximately linear relationship. Interestingly, a significantly positive association of 2-hPBG-FBG with eGFR was observed in all three models for the newly diagnosed diabetes patients with HbA1c<7% only, which was independent of age, gender, BMI, systolic BP, diastolic BP, fasting insulin, cholesterol, triglycerides, smoking, drinking and HbA1c. Moreover, we found a nonlinear association of 2-hPBG-FBG and eGFR using a generalized additive model after multivariable adjustment (*P*<0.001; Fig. 1). In contrast, a negative relationship between HbA1c and eGFR was observed in the patients with HbA1c (%) ≥7.

**Table 2:** Multiple linear regression analysis of association of 2-hPBG-FBG with eGFR.

		HbA1c (%) <7		HbA1c (%) ≥7	
Model	Independent variable	β Coefficient (95% CI)	*P*-value	β Coefficient (95% CI)	*P*-value
Model 1	2-hPBG-FBG, per mg/dL	0.041 (0.010 to 0.072)	**0.009**	−0.026 (−0.061 to 0.009)	0.146
Model 2	2-hPBG-FBG, per mg/dL	0.040 (0.019 to 0.061)	**<0.001**	−0.010 (−0.035 to 0.015)	0.419
Model 3	2-hPBG-FBG, per mg/dL	0.036 (0.015 to 0.057)	**0.001**	0.010 (−0.014 to 0.037)	0.492
	HbA_1c_, per % unit	−1.272 (−3.424 to 0.880)	0.246	−0.779 (−1.547 to −0.011)	**0.047**

Model 1: not adjusted; Model 2: adjusted for age, gender, BMI, systolic BP and diastolic BP; Model 3: Model 2 plus Log (fasting insulin), cholesterol, Log (triglycerides), smoking and drinking.

### 2-hPBG-FBG is closely associated with increased risk of GHF in newly diagnosed diabetes patients with HbA1c<7%

As shown in Table 3, we analyzed the association of increased 2-hPBG-FBG with the risk of GHF for the three models. As expected, 2-hPBG-FBG significantly increased the risk of GHF in the newly diagnosed diabetes patients with HbA1c<7% but not in the subjects with HbA1c≥7%. In model 1, a 38.2% increase in GHF risk for every 36 mg/dl (2.0 mmol/L) 2-hPBG-FBG increase was observed. After adjusting for age, gender, BMI, systolic BP and diastolic BP, the GHF risk increased by 61.4% for every 36 mg/dl (2.0 mmol/L) 2-hPBG-FBG increase. Further adjustments for fasting insulin, cholesterol, triglycerides, smoking, drinking and HbA1c revealed a 64.9% increase in the GHF risk. In contrast, HbA1c (an indicator of average blood glucose) was not associated with an increased risk of GHF.

**Table 3:** Multiple logistic regression analysis of association of 2-hPBG-FBG with GHF.

		HbA1c (%) <7		HbA1c (%) ≥7	
Model	Independent variable	Odds ratio (95% CI)	*P*-value	Odds ratio (95% CI)	*P*-value
Model 1	2-hPBG-FBG, per 36 mg/dL	1.382 (1.051 to 1.818)	**0.021**	0.520 (0.333 to 0.814)	**0.004**
Model 2	2-hPBG-FBG, per 36 mg/dL	1.614 (1.068 to 2.440)	**0.023**	0.721 (0.372 to 1.396)	0.332
Model 3	2-hPBG-FBG, per 36 mg/dL	1.649 (1.061 to 2.565)	**0.026**	0.742 (0.347 to 1.589)	0.442
	HbA_1c_, per % unit	0.889 (0.299 to 2.638)	0.831	0.928 (0.606 to 1.421)	0.731

Model 1: not adjusted; Model 2: adjusted for age, gender, BMI, systolic BP and diastolic BP; Model 3: Model 2 plus fasting insulin, cholesterol, triglycerides, smoking and drinking.

## Discussion

Because GHF is a phenomenon occurring early in the clinical course of diabetes that may lead to the development of DN due to its associated glomerular damage, it is crucial to screen for risk factors of GHF during the early stage of diabetes. Therefore, we selected newly diagnosed diabetes patients as our study population. In addition, to further subdivide these patients, they were placed into two groups based on their HbA1c values. Interestingly, we found that the fluctuations between the fasting and 2-hPBG-FBG states were closely associated with eGFR and the increased risk of GHF in the patients with very early-stage diabetes only (HbA1c<7%), which indicated the importance of glucostasis between the fasting and 2-h postload states. This association was not observed for those patients with poorly controlled diabetes, which may have been because for newly diagnosed diabetics, HbA1c may be essentially reflective of the clinical course of diabetes. Those patients with higher HbA1c levels may have had diabetes for a longer period of time and thus may not have been at the very early stage of the disease. Because GHF is always present at the very early stage of diabetes, those patients with higher HbA1c levels may not have possessed GHF any longer. In contrast, they may have presented with decreased eGFR.

The two main risk factors for DN are hyperglycemia and arterial hypertension [24]. Therefore, we adjusted for HbA1c, which is reflective of the average blood glucose level within the past two to three months, in addition to BP to analyze the association of blood glucose fluctuations with GHF. The traditional risk factors for DN also include sex, age, smoking, drinking, dyslipidemia and insulin resistance [24]. Thus, we further adjusted for the above risk factors in model 3 to verify the relationship between blood glucose fluctuations and GHF. Notably, because the calculations of HOMA-IR and 2-hPBG-FBG both involve FBG, we added fasting insulin rather than HOMA-IR to model 3. Interestingly, the GHF risk increased by 64.9% for every 36 mg/dl (2.0 mmol/L) 2-hPBG-FBG increase after adjusting for age, gender, BMI, systolic BP, diastolic BP, fasting insulin, cholesterol, triglycerides, smoking, drinking and HbA1c. In contrast, average blood glucose (HbA1c) was not associated with GHF.

Two recent studies have revealed that HbA1c fluctuation independently affects the development of CKD in type 2 diabetes patients [16], [17], reflecting the effects of long-term glycemic fluctuations on renal complications. The Renal Insufficiency And Cardiovascular Events (RIACE) Italian multicenter study [16] has shown that in patients with type 2 diabetes, HbA1c fluctuations affect (albuminuric) CKD more than average HbA1c levels. In addition, a study [17] conducted in Hong Kong has found that long-term glycemic fluctuations as expressed by the SD of HbA1c predict the development of renal and cardiovascular complications. Although the effects of long-term glycemic fluctuations on renal complications have been investigated in some studies, population studies of the influences of short-term glycemic fluctuations, and particularly those of glucose fluctuations, between the fasting and 2-h postload states on renal function are scarce, and few studies have been conducted using animal models, which have shown that blood glucose fluctuations may induce renal pathological changes in diabetic rodents [25], [26]. Blood glucose fluctuation may accelerate the development of kidney fibrosis in diabetic mice by increasing collagen production and inhibiting collagen degradation, and both the ERK/MAPK and TGF-β/Smad signaling pathways seem to play roles in its development, leading to changes in the GFR [25]. Moreover, a more serious degree of glomerular sclerosis has been observed in association with blood glucose fluctuations in mice compared with those with sustained high blood glucose levels [26]. The influences of glucose fluctuations on endothelial function have also been explored [27], [28]. Glucose fluctuations may induce the production of reactive oxygen species (ROSs) in endothelial cells, facilitating the development of vascular endothelial dysfunction in rats with type 2 diabetes [27], [28]. In the present study, we also observed that glucose fluctuations led to the increased risk of GHF during the early stage of diabetes.

Creatinine-based equations for estimating the GFR include the Cockcroft-Gault equation, the Modification of Diet in Renal Disease (MDRD) equation and the CKD-EPI equation. Currently, the Cockcroft-Gault equation has been supplanted by the latter two equations [29]. In addition, the CKD-EPI equation is more accurate and precise in estimating the GFR than the MDRD equation in middle-aged and older South Asians [30]. Therefore, we selected the CKD-EPI equation to calculate the eGFR.

Our study contains some limitations. First, a cross-sectional study cannot infer causality between 2-hPBG-FBG and GHF. Second, fluctuations between the fasting and 2-h postload glucose (2-hPBG-FBG) states do not precisely reflect glucose fluctuations. However, as a simple indicator of glucose fluctuations, 2-hPBG-FBG may be easily applicable in clinical practice. Third, our study contained only middle-aged and older Chinese people; age and ethnic differences should be taken into account when assessing these results. Fourth, we diagnosed diabetes based on one 75-g OGTT, which should be repeated and confirmed. Finally, the GFR based on creatinine levels and estimated by the CKD-EPI equation may not accurately reflect kidney function, which may have affected the accuracy of the GHF estimation. However, the gold standard method for measuring the GFR (the isotope clearance measurement) is very expensive and time-consuming, so the use of creatinine-based equations to estimate the GFR is logical for large epidemiological studies.

In conclusion, we found that fluctuations between the fasting and 2-hPBG-FBG states are associated with an increased risk of GHF in newly diagnosed diabetes patients with HbA1c<7% but not in subjects with HbA1c≥7%. For newly diagnosed diabetics, HbA1c as well as 2-hPBG-FBG should be strictly controlled to prevent diabetes complications. In addition, longitudinal studies are needed to explore the extent to which the 2-hPBG-FBG value should be controlled in clinical practice to effectively aid in the prevention of DN.
